# Influence of Ecological Factors on the Production of Active Substances in the Anti-Cancer Plant *Sinopodophyllum hexandrum* (Royle) T.S. Ying

**DOI:** 10.1371/journal.pone.0122981

**Published:** 2015-04-15

**Authors:** Wei Liu, Jianjun Liu, Dongxue Yin, Xiaowen Zhao

**Affiliations:** College of Forestry, Northwest A & F University, Yangling, China

## Abstract

The quality of traditional Chinese herbal medicine, which plays a very important role in the health system of China, is determined by the active substances produced by the plants. The type, content, and proportion of these substances may vary depending on ecological factors in areas where the plants are grown. *Sinopodophyllum hexandrum* (Royle) T.S. Ying, an endangered plant species with great medical value, was investigated in eight production locations representative of its natural geographical distribution range in China. The correlation between the contents of the active ingredients extracted from the roots and rhizomes of *S*. *hexandrum* and the ecological factors were evaluated step-by-step using a series of computational biology methodologies. The results showed that ecological factors had significant effects on the contents but not on the types of the active ingredients in eight production locations. The primary ecological factors influencing the active substances included the annual average precipitation, July mean temperature, frost-free period, sunshine duration, soil pH, soil organic matter, and rapidly available potassium in the soil. The annual average precipitation was the most important determinant factor and was significantly and negatively correlated with the active ingredient contents (*P* < 0.001). In contrast, organic matter was the most important limiting factor and was significantly and positively correlated with the active substances. These ecological factors caused 98.13% of the total geographical variation of the active ingredient contents. The climate factors contributed more to the active ingredient contents than did the soil factors. It was concluded that from the view of the contents of the secondary metabolites and ecological factors of each growing location, in Jingyuan, Ningxia Province, and Yongdeng, Gansu Province, conditions were favorable to the production of podophyllotoxin and lignans, whereas in Shangri-La, Yunnan Province, and Nyingchi, Tibet, conditions were favorable to the production of quercetin and kaempferol.

## Introduction

Plants have been used for centuries in traditional medicine and currently a quarter of all prescribed pharmaceuticals in industrialized countries contain compounds derived from plants, directly or indirectly via semi-synthesis [1]. Traditional Chinese herbal medicine (TCHM) has played important role in the Chinese medical system [2]. *Sinopodophyllum hexandrum* (Royle) T.S. Ying, family Berberidaceae, the only species of this genus in China, commonly known as Himalayan mayapple, is an important and rare TCHM native to the Himalayan regions [3]. The roots and rhizomes of *S*. *hexandrum* contain large amounts of lignans, three times the amount of lignans produced by the relative American species *Podophyllum peltatum* [4–6]. The most important lignan for human health is arguably the most active cytotoxic aryltetralin lignan, podophyllotoxin (PTOX), as a precursor for the semi-synthesis of the anticancer pharmaceuticals etoposide (VP-16), teniposide (VM-26), GP-7, and NK-611 [5,6,7–10]. These chemotherapeutic drugs have been used for cancer therapy, including the treatment of lung cancer, cervical cancer, testicular cancer, neuroblastoma, hepatoma, and certain leukemias [5,6,11,12]. In addition to its role in anti-cancer drugs, PTOX is the starting compound of a new derivative, CPH-82, which is included in clinical trials for the treatment of rheumatoid arthritis in Europe [13].

In addition to lignans, flavonoids such as quercetin and kaempferol have also been found in *Sinopodophyllum hexandrum* [14,15]. Studies have shown that flavonoids have multiple biological effects, including antioxidant [16], anti-inflammatory, antiviral, and platelet aggregation inhibition [17]. Some reports have suggested that flavonoid supplements could prevent cardiovascular diseases [16,18,19]. Due to their medicinal importance, wild *S*. *hexandrum* have been extensively harvested in the alpine Himalayan regions as the commercial source of PTOX and flavonoids. The extensive harvest of these plants added *S*. *hexandrum* to the endangered species list of the Convention on International Trade in Endangered Species of Wild Fauna and Flora [20,21]. *S*. *hexandrum* has been classified as an endangered species (grade 3) since 1987 and catalogued in the Chinese Plant Red Book [22].

Currently, with the enhanced awareness of its medicinal value and superior efficacy in clinical applications, the availability of PTOX from plants has become increasingly limited due to intense collection, habitat fragmentation, and the lack of organized cultivation in China. Wild *Sinopodophyllum hexandrum* populations could become extinct without rational exploitation measures, costing humans an ideal drug against cancer. Knowledge of the optimization of production locations is an important prerequisite for a rational exploitation program planning. However, previous studies have focused on the determination and separation of the phytochemicals [5,14,15,23–27], pharmacological functions [28–32], genetic diversity [33,34], micropropagation [35], and system evolution [36]. Studies on the optimization of production locations are limited despite the fact that ecological factors highly affect the secondary metabolite synthesis and their contents [37–39]. The metabolism and accumulation of active ingredients, most of which are secondary metabolites, is the reflection of integrated influences of multiple ecological factors on the medicinal plant during their developmental and growth periods in addition to genetic factors. Certain metabolites are only synthesized under specific environments, or their contents significantly increase under specific environments [40]. Furthermore, previous studies have demonstrated that medicinal plants grown in different environments produce different contents of secondary metabolites, resulting in differences in their medicinal qualities [37]. Traditionally, the qualities of TCHMs were assessed by their external appearance, and by the experiences of medicinal practice of ancient physicians. In this process, the concept of so called geo-authentic herbal drugs was established. Most geo-authentic traditional herbs produced in their native geographical area contain adequate effective chemical constituents. For example, only *Neopicrorhiza scrophulariiflora* (Pennell) D.Y. Hong, one of the well-known herbal drugs in TCHM, produced in Tibet, China is officially recognized for use in medicinal practice [41]. By contrast, only *Panax ginseng* C.A. Mey., produced in northeastern China is officially recognized as medicinal drugs [41]. For *Glycyrrhiza uralensis* Fisch. ex DC. and *Astragalus membranaceus* Bunge, however, only those produced in Inner Mongolia Autonomous Region, China are officially recognized as medicinal resources [41].

The existing variations in podophyllotoxin contents among the *Sinopodophyllum hexandrum* populations were proved to be coupled with geographical altitude and local ecological conditions (temperature, rainfall, humidity, soil pH, etc.) but not with genetic basis in several studies [42,43]. Hence, we investigated the correlation between the main active ingredients including podophyllotoxin (Y_P_), 4’-demethylpodophyllotoxin (Y_DP_), 4’-demethylepipodophyllotoxin (Y_DEP_), total lignans (Y_TL_), quercetin (Y_Q_) and kaempferol (Y_K_) of *S*. *hexandrum* from representative production regions throughout China and ecological factors (soil and climate factors). The present study aims at clarifying the environmental factors affecting the production of active ingredients of *S*. *hexandrum* in different production locations where *S*. *hexandrum* has been naturally growth according to historical records in order to suggest the best production areas for this wild species, promote its reasonable exploitation for the production of medicinal drugs rather than extensively harvesting wild resources.

## Materials and Methods

### Ethics statement

The research activities were scientifically conducted under the permits issued by the local forestry departments. A detailed description of the material collection procedures was provided by the authors in S1 Text. All locations where the plants were collected were not privately owned. The experimental procedures were approved by the Ethics Committee for Plant Experiments of Northwest A & F University (20120712004) and the State Forestry Administration, P. R. China. The names of the authorities that issued the permit for each location were listed in S1 Table.

### Plant materials and soil samples

**Fig 1:**
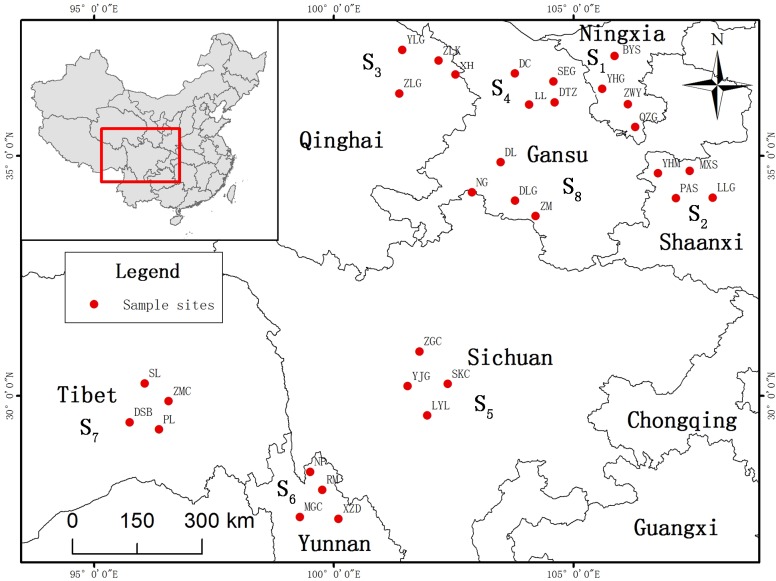
Growing locations of the 32 *S*. *hexandrum* populations involved in seven provinces of China sampled for this study. Maps generated using ArcGIS 10.0 (ESRI Inc. 2014).

*Sinopodophyllum hexandrum* distribution pattern and extent of in China were investigated from 2010 to 2011. According to the field survey and species information in Flora of China [3], *S*. *hexandrum* have a natural distribution in seven provinces of China: Ningxia Province, Shaanxi Province, Qinghai Province, Gansu Province, Sichuan Province, Yunnan Province and Tibet. *S*. *hexandrum* roots and rhizomes were collected from eight representative production locations (Jingyuan in Ningxia Province, Mei county in Shaanxi Province, Huzhu in Qinghai Province, Yongdeng in Gansu Province, Kangding in Sichuan Province, Shangri-la in Yunnan Province, Nyingchi in Tibet and Diebu in Gansu Province) that encompassed all of its natural distribution areas involved in seven provinces of China (Fig 1) from July 19, 2012, to September 17, 2012, to ensure the consistency of the collection period. Four natural populations that were similar in growth statues (each population was separated geographically by at least 30 km) were selected at each test location. 20 healthy individuals of each natural population were collected. The distance between the adjacent individuals was at least 5 m to increase the likelihood of sampling inter-individual variations within each population [33,34]. A total of eighty individual samples were collected at each test location, as shown Table 1. Root and rhizome samples were picked up respectively from four directions (north, south, east, and west) of three positions (up, middle and low part) of the hypogeal part of each individual and then mixed as one individual sample. In each study site, eighty individual samples of four populations were evenly mixed as a test sample [37]. Eight test samples obtained from eight study sites were dried under the vacuum at 40°C, and ground into powders, by which the contents of the active ingredients were measured. Simultaneously, soil rhizosphere were collected and treated for each study site, thereby obtained eight soil samples from eight study sites for the measurement of soil factors. Voucher specimens from all populations were identified by professor Jianjun Liu of Northwest A&F University and were deposited at the Herbarium of Northwest A&F University (WUK0780759-0780790).

**Table 1:** Sample information of the 8 sampling sites in seven provinces of China.

No.	Locations	Population	Code	Coordinates	N	Altitude (m)	Soil type	Climate zones
S_1_	Jingyuan, Ningxia	Baiyunshan	BYS	E106°15′N35°37′	20	2232	Gray cinnamonic soil	Humid and semi-humid temperate climate zone
		Yehegu	YHG	E106°13′N35°31′	20	2370	Gray cinnamonic soil	Humid and semi-humid temperate climate zone
		Zhiwuyuan	ZWY	E106°18′N35°22′	20	2080	Gray cinnamonic soil	Humid and semi-humid temperate climate zone
		Qiaozigou	QZG	E106°22′N35°15′	20	2564	Gray cinnamonic soil	Humid and semi-humid temperate climate zone
S_2_	Mei county, Shaanxi	Pingansi	PAS	E107°43′N34°1′	20	2815	Dark brown soil	Semi-humid warm temperate continental monsoon climate
		Mingxingsi	MXS	E107°44′N34°0′	20	2637	Dark brown soil	Semi-humid warm temperate continental monsoon climate
		Yuhuangmiao	YHM	E107°22′N34°5′	20	1780	Dark brown soil	Semi-humid warm temperate continental monsoon climate
		Liulingou	LLG	E108°10′N33°52′	20	1013	Dark brown soil	Semi-humid warm temperate continental monsoon climate
S_3_	Huzhu, Qinghai	Zhalongkou	ZLK	E102°34′N36°53′	20	2264	Alpine meadow soil	Semi-arid continental plateau monsoon climate zone
		Zhalonggou	ZLG	E102°37′N36°47′	20	2698	Alpine meadow soil	Semi-arid continental plateau monsoon climate zone
		Yuanlongogu	YLG	E102°27′N36°54′	20	3069	Alpine meadow soil	Semi-arid continental plateau monsoon climate zone
		Xiahe	XH	E102°42′N36°44′	20	3169	Alpine meadow soil	Semi-arid continental plateau monsoon climate zone
S_4_	Yongdeng, Gansu	Suoergou	SEG	E102°43′N36°40′	20	2389	Alpine meadow soil	Semi-arid continental cold temperate climate zone
		Lalagou	LL	E102°43′N36°35′	20	2733	Alpine meadow soil	Semi-arid continental cold temperate climate zone
		Dachang	DC	E102°44′N36°44′	20	2449	Alpine meadow soil	Semi-arid continental cold temperate climate zone
		Datanzigou	DTZ	E102°46′N36°33′	20	2530	Alpine meadow soil	Semi-arid continental cold temperate climate zone
S_5_	Kangding, Sichuan	Yajaigeng	YJG	E101°57′N30°0′	20	2946	Rich in humus loam	Humid subtemperate plateau climate zone
		Laoyulin	LYL	E101°59′N29°55′	20	3788	Rich in humus loam	Humid subtemperate plateau climate zone
		Shengkangcun	SKC	E102°1′N30°4′	20	3207	Rich in humus loam	Humid subtemperate plateau climate zone
		Zhonggucun	ZGC	E101°54′N30°16′	20	3554	Rich in humus loam	Humid subtemperate plateau climate zone
S_6_	Shangri-la, Yunnan	Rime	RM	E99°37′N27°51′	20	3528	Subalpine shrub soil	Mountains cool temperate monsoon climate zone
		Naipi	NP	E99°36′N28°2′	20	3432	Subalpine shrub soil	Mountains cool temperate monsoon climate zone
		Xiaozhongdian	XZD	E99°56′N27°28′	20	3590	Subalpine shrub soil	Mountains cool temperate monsoon climate zone
		Mugaocun	MGC	E99°34′N27°30′	20	2250	Subalpine shrub soil	Mountains cool temperate monsoon climate zone
S_7_	Nyingchi, Tibet	Zhangmaicun	ZMC	E94°20′N29°40′	20	3097	Subalpine shrub soil	Temperate continental plateau climate zone
		Selong	SL	E94°11′N29°44′	20	3173	Subalpine shrub soil	Temperate continental plateau climate zone
		Pula	PL	E94°22′N29°27′	20	3256	Subalpine shrub soil	Temperate continental plateau climate zone
		Duosongba	DSB	E94°13′N29°37′	20	3855	Subalpine shrub soil	Temperate continental plateau climate zone
S_8_	Diebu, Gansu	Zemo	ZM	E103°21′N33°45′	20	2728	Alpine meadow soil	Mountain continental climate zone
		Dalong	DL	E103°14′N35°2′	20	2620	Alpine meadow soil	Mountain continental climate zone
		Dalagou	DLG	E103°22′N33°52′	20	2677	Alpine meadow soil	Mountain continental climate zone
		Nagai	NG	E103°14′N33°51′	20	2963	Alpine meadow soil	Mountain continental climate zone

**Note**: N means sample size.

### Related data of ecological factors

Soil samples were used to determine the key soil factors (soil parameters) including rapidly available nitrogen (X_RAN_), rapidly available phosphorus (X_RAP_), rapidly available potassium (X_RAPM_), total nitrogen (X_TN_), total phosphorus (X_TP_), total potassium (X_TPM_), organic matter (X_OM_) and pH (X_pH_) using the modified soil chemistry analysis methods described by Sparks *et al*. [44]. Related data of climate factors including january average temperature (X_JAT_), july mean temperature (X_JMT_), annual accumulated temperature(≥10ଌ) (X_AAT_), annual highest temperature (X_AHT_), annual lowest temperature (X_ALT_), annual mean temperature (X_AMT_), annual average precipitation (X_AAP_), annual sunshine duration (X_ASD_), frost free period (X_FFP_) and relative humidity (X_RH_) in the recent 50 years (1963 ~ 2012) were also collected from local meteorological bureaus (stations) for the eight study sites. The names of meteorological bureaus (stations) for each location were provided in S2 Table.

The soil factors and climate factors of the eight study sites were summarized in Table 2.

**Table 2:** Main ecological factors of eight locations throughout China.

Itemss	S_1_	S_2_	S_3_	S_4_	S_5_	S_6_	S_7_	S_8_
X_RAN_	6.31±0.03 abcd	28.12±0.02 cde	32.65±0.04 abc	8.09±0.07 ab	7.43±0.04 abc	8.13±0.03 abcd	32.21±0.02 cde	35.82±0.03 abc
X_RAP_	6.42±0.06 ab	7.58±0.05 a	8.56±0.02 b	11.37±0.15 de	8.25±0.24 ab	7.24±0.06 ab	8.85±0.05 a	10.13±0.02 b
X_RAPM_	107.69±0.02 cde	357.53±0.04 e	96.71±2.14 de	132.04±4.30 e	160.41±0.01 de	135.96±0.02 cde	384.35±0.03 e	112.71±0.04 de
X_TN_	0.23±0.02 e	0.25±0.001 bc	0.20±0.004 e	0.29±0.005 de	0.32±0.004 e	0.17±0.001 e	0.33±0.001 bc	0.16±0.005 e
X_TP_	0.03±0.001 ab	0.11±0.002 cde	0.14±0.003 e	0.21±0.005 bc	0.05±0.001 de	0.02±0.001 ab	0.15±0.002 cde	0.32±0.003 e
X_TPM_	2.45±0.01 e	1.49±0.02 de	1.62±0.04 de	1.77±0.03 abc	2.46±0.05 e	2.60±0.01 e	1.56±0.02 de	2.35±0.04 de
X_OM_	5.00±0.01 bcde	6.52±0.01 bcd	5.40±0.01 b	10.02±0.06 de	6.38±0.03 cde	4.09±0.01 bcde	7.22±0.03 bcd	6.50±0.01 b
X_pH_	6.20±0.05 abc	7.31±003 bcd	6.59±0.05 cde	6.39±0.07 cde	6.12±0.06 abcd	6.40±0.05 abc	7.09±003 bcd	6.20±0.05 cde
X_JAT_	-7.0±0.01 bcd	-4.0±0.04 abcd	-8.2±0.02 de	-7.6±0.05 bcd	-2.2±0.04 bcd	-5.6±0.01 bcd	1.5±0.03 abcd	-5.0±0.02 de
X_JMT_	19.0±0.01 abc	22.5±0.07 ab	23.4±0.03 bcd	21.7±0.04 d	15.5±0.01 ab	13.4±0.02 abc	16.3±0.03 ab	16.8±0.05 bc
X_AAT_	1847.43±0.03 bc	2390.80±0.05 ab	3643.87±0.06 c	4108.22±0.04bcd	1265.20±0.04bcde	1526.70±0.03 bc	2263.20±0.05 ab	1931.52±0.03 bcd
X_AHT_	40.44±0.08 cd	32.80±0.04 bc	39.50±0.01 abc	35.00±0.03 ab	31.70±0.01 cd	24.60±0.04 cd	30.60±0.05 bc	33.90±0.02 abc
X_ALT_	-24.00±0.04 de	-25.50±0.06 d	-26.62±0.03 bcd	-24.24±0.01 bc	-28.91±0.04 abc	-23.40±0.04 de	-14.4±0.02 d	-20.30±0.03bcd
X_AMT_	6.90±0.05 ab	7.60±0.08 d	4.32±0.04 e	6.40±0.02 e	7.00±0.05 cde	5.50±0.05 ab	9.00±0.08 d	6.80±0.04 e
X_AAP_	616.32±0.01 abc	635.44±0.04 bc	620.30±0.04abc	310.27±0.06 cde	880.30±0.04 ab	627.20±0.01 abc	650.45±0.04 bc	642.41±0.06abc
X_ASD_	2370.12±0.04 ab	2021.35±0.07bcde	1226.55±0.03bc	2659.62±0.06abc	1738.34±0.03 a	2206.40±0.04 ab	2020.53±0.07bcde	2246.20±0.01bc
X_FFP_	132.61±15.38abc	158.00±9.66 a	230.54±11.55ab	121.52±8.12 abc	180.00±1.23 a	167.00±15.38abc	180.00±9.66 a	147.35±11.55ab
X_RH_	63±0.02 ab	70±0.04 a	74±0.05 c	55±0.02 bc	68±0.01 ac	45±0.02 a	55±0.03 ab	65±0.04 b

**Note:** For soil factors and climate factors, values were given as mean ± SD. For each location, values followed by the same small letter did not share significant differences according to Duncan’s test (*P* < 0.05). X_RAN_(mg/kg)-Rapidly available nitrogen, X_RAP_(mg/kg)-Rapidly available phosphorus, X_RAPM_(mg/kg)-Rapidly available potassium, X_TN_(%)-Total nitrogen, X_TP_(%)-Total phosphorus,X_TPM_(%)-Total potassium, X_OM_(%)-Organic matter, X_pH_-pH, X_JAT_(°C)-January average temperature, X_JMT_(°C)-July mean temperature, X_AAT_(°C)-Anaual accumulated temperature(≥10°C), X_AHT_(°C)-Annual highest temperature, X_ALT_(°C)-Annual lowest temperature, X_AMT_(°C)-Anaual mean temperature, X_AAP_(mm)-Anaual average precipitation, X_ASD_(h)-Anaual sunshine duration, X_FFP_(d)-Frost free period, X_RH_(%)-Relative humidity.

### Sample preparation

First, 5.0 g of pulverized roots and rhizomes of *Sinopodophyllum hexandrum* was extracted by refluxing three times (2 h each time) at 80°C with 125 mL of ethanol. The extraction solutions were combined and then subjected to ultrasonic vibration for 30 min. After cooling to room temperature, the obtained solutions were filtered, concentrated into red brown extracts by evaporation, reflux-extracted twice (2 h each) with benzene, and then filtered. The filtered liquor was concentrated for crystallization, yielding 125 mg of light-yellow crystals. Subsequently, the crystalline substances were dissolved, and diluted with methanol to produce a stock solution (1 mg/mL). 2 mL of the stock solution was filtered through a 0.22 μm membrane filter, and an aliquot of each filtrate (10 μL) was directly injected into the liquid chromatography (LC) instrument for analysis.

### Chromatographic analysis by reverse phase-high performance liquid chromatography (RP-HPLC)

An LC system consisting of a vacuum degasser, quaternary pump, and variable wavelength detector (VWD) detector (Agilent Co., USA) was used to acquire the chromatograms and UV spectra [15]. Chromatographic separation was achieved using a C18 column (250×4.6 mm, 5 μm; Diamonsil, USA), and a gradient solvent system comprised of 0.1% formic acid-water (solvent A) and acetonitrile (solvent B). The gradient profile was as follows: 0 min–25 min, linear 10%–35% of B; 25 min–55 min, linear 35%–65% of B; 55 min–85 min, linear 65%–75% of B; 85 min–100 min, linear 75%–100% of B, 100 min–110 min, equicontinuous 100% of B. A flow rate of 0.8 mL/min was used. UV detection was carried out at 290 nm. After 110 min, no peaks were found; chromatograms within 110 min were studied. The standard solutions including PTOX, 4-demethylpodophyllotoxin, 4-demethylepipodophyllotoxin, quercetin, and kaempferol were used for the calibration of a standard curve using an external standard method. The analyses were performed in triplicate.

The cosine values of the vectorial angle of the entire chromatographic patterns among the samples were calculated and the simulative mean chromatogram was calculated using the Computer Aided Similarity Evaluation System (CASES) software for the chromatographic fingerprinting of TCHM. This software was employed to synchronize the chromatographic peaks, to calculate the cosine values of the vectorial angle among different chromatograms, as well as to compute the mean chromatogram as a representative standard chromatogram for a group of chromatograms. The cosine values of the two chromatograms approaching 1 indicates that they are highly similar. The standard LC fingerprint was set up with the median of all chromatograms [45–47]. The similarities of the entire chromatographic profiles were analyzed among the tested samples. The contents of PTOX, 4-demethylpodophyllotoxin, 4-demethylepipodophyllotoxin, quercetin, and kaempferol were respectively obtained according to the relative retention time and relative peak area of reference chromatographs of the standards with the help of the standard curve equation.

### Measurement of total lignans

A modified UV spectrophotometry method (reference wavelength method) was used to determine the total lignan contents [48]. 4 mL of the diluted solution (1 mg/mL) prepared as described in the “Sample preparation” subsection was pipetted and then transferred to a 10 mL volumetric flask and made up to the mark with methanol. The absorbance at 290 nm and 392 nm was measured against a blank (100% of methanol), respectively. Standard PTOX (5.25–95.40 mg/L) and kaempferol (5–60 mg/L) products were used for the standard curve calibration. All measurements were repeated in triplicate.

### Data analyses

The results were presented as the mean value ± standard deviation (SD). The data were analyzed by one-way ANOVA followed by Duncan multiple comparison based on the SPSS 19.0 software. Values of *P* < 0.05 were considered statistically significant. Chromatographic analysis of eight plant samples (S_1_-S_8_, Table 1) from eight study sites was conducted by CASES, a chemometrics computer software for the chromatographic fingerprinting of TCHM developed by the Institute of Pharmacy Engineering (Zhejiang University, Hangzhou, China) and endorsed by the National Pharmacopoeia Committee of the People’s Republic of China for similarity studies of the chromatographic fingerprints of Chinese herbs.

Prior to the analysis, spatial autocorrelation (given as global Moran’s I) were examined across realisations using the software Geoda 1.0.0 (http://geodacenter.asu.edu/software/downloads) [49], no spatial autocorrelation existed in present study region (global Moran’s I values = 0.000±0.000, *P*>0.1). Five methodologies were performed step-by-step to analysis systematically the influence of ecological factors on the active ingredient contents of roots and rhizomes of *Sinopodophyllum hexandrum*, which also represented a quantitative comparative analysis of the dynamic process of plant and the environment. Correlation analysis (CA) and principal component analysis (PCA) were carried out using SPSS 19.0 software. CA attempted to test whether a correlation between active ingredients and the ecological factors or not to provide a basis for subsequent statistical analysis. PCA has recently become the tool of choice for monitoring complex chemical processes [50–53]. PCA has the strong advantage of significantly reducing the dimension of the complex components of plants while preserving most of the variance within by using dependencies among large numbers of variables without requiring knowledge of the data set in order to visualize high dimensional data and identify the most important variables [54]. PCA in the present study was used to find out principal components of active ingredients. Gray correlation analysis (GCA), a system analysis technique, is usually applied in indefinite gray systems, including those with small sampling and poor data information, in which limited information is available, others are not known [37,55,56]. Path analysis (PA) deals with the quantitative relationship between dependent and independent variables to explain the relative significance of each factor to the dependent variables [37,57]. GCA and PA were conducted by DPS 2006 software to select the primary ecological factors and evaluate correlations between the active ingredients and these primary ecological factors, respectively. The correlation coefficient in PA contains the direct path coefficient and the indirect path coefficient. However, a large direct effect between independent variables and dependent variables does not always imply a strong correlation between them. Therefore, PA could not identify which independent variable has the decisive effect on the dependent variable. Thus, further analysis using the decisive coefficient (analysis of decisive degree (ADD)) should be conducted to reflect the comprehensive decisive effect of independent variables on dependent variables by other independent variables and determine the determinant factors [58]. Ecological factors were used as independent variables and active ingredients were used as dependent variables in the each test.

## Results

### Validation of the HPLC procedure

**Fig 2:**
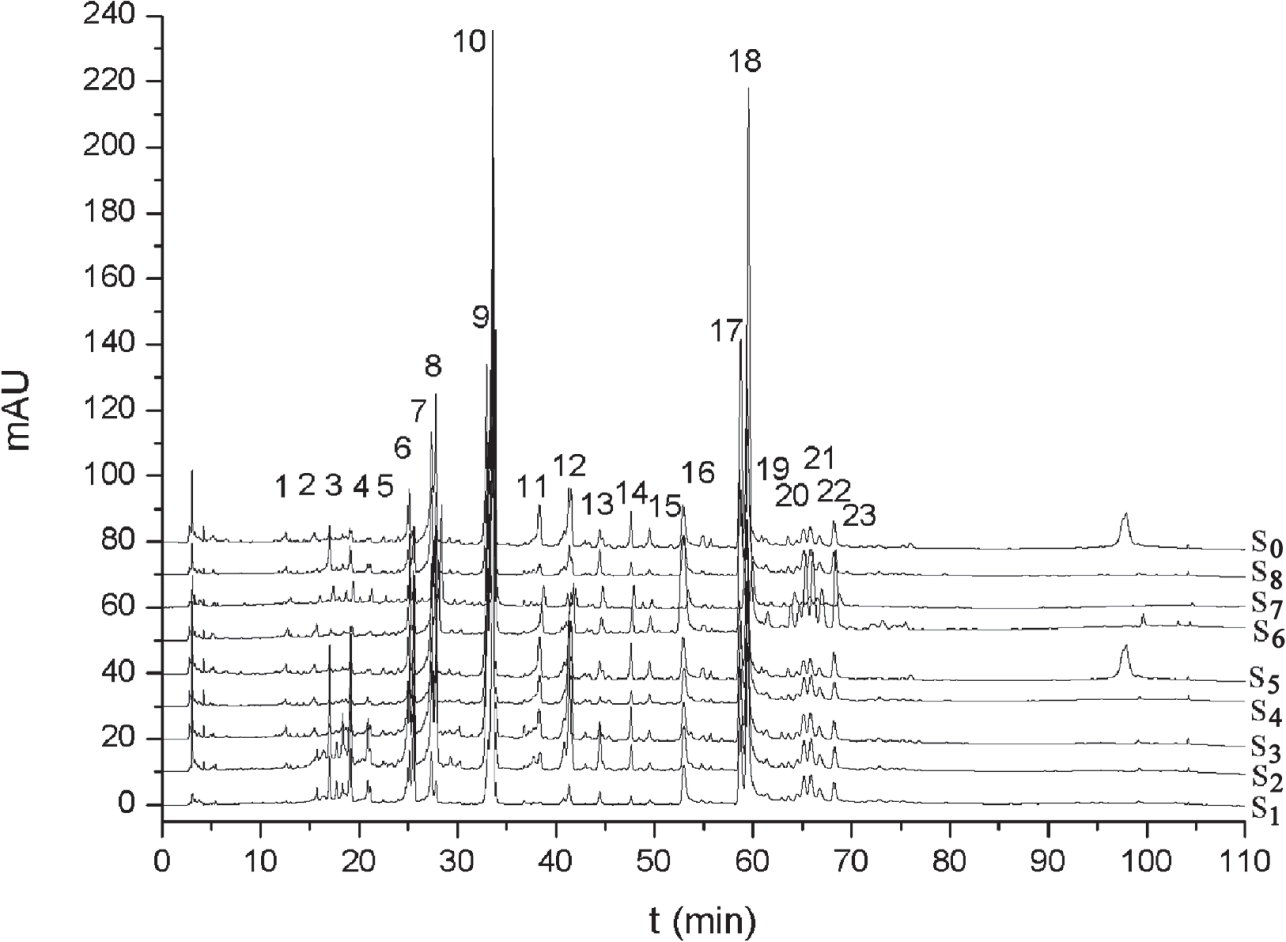
Chromatography of all the samples from eight different production locations (S_1_-S_8_). S_1_: Jingyuan, Ningxia; S_2_: Mei county, Shaanxi; S_3_: Huzhu, Qinghai; S_4_: Yongdeng, Gansu; S_5_: Kangding, Sichuan; S_6_: Shangri-la, Yunnan; S_7_: Nyingchi, Tibet; S_8_: Diebu, Gansu.

Method reproducibility and repeatability of the method were evaluated by analyzing six injections of the same sample solution and six replicates of sample extraction, respectively. Precision of retention times and peak areas of the five compounds 4’-Demethylpodophyllotoxin (peak 7), 4’-Demethylepipodophyllotoxin (peak 8), podophyllotoxin (peak 10), kaempferol (peak 14), quercetin (peak 15) (Fig 2) for replicated injection were in the range of 0.16–1.4% and 1.3–3.1% of relative standard deviation (RSD) (n = 6), respectively. The RSD of peak area of the five compounds in sample replicates were about 2.3–4.1% (n = 6). To confirm the accuracy of the method, a recovery experiment was performed by mixing quantified samples with specific quantities of standard compounds. The average percentages of recovery of the five compounds ranged from 98.25±5.27% to 103.61±3.12%. In addition, the RSD varied from 2.73% to 5.38% (n = 6). The limit of detection (LOD) (signal/noise = 3) and the limit of quantification (LOQ) (signal/noise = 10) of the five compounds varied within the range 1.93–3.07 ng/mL and 7.86–10.23 ng/mL. The stability of the five compounds in the sample solution was evaluated by determining their RPA after storage for 0–24 h, respectively. The RSDs of the retention times and peak areas were both less than 3%. All the results demonstrated that the conditions for the fingerprint analysis were repeatable and accurate (Table 3).

**Table 3:** Validation test for the quantitative determination of the five compounds (n = 6).

Peak No.	Compound	Linearity range (μg/mL)	Calibration curve	Correlation coefficient	LOD (ng/mL)	LOQ(ng/mL)	Recovery experiment	
							Average recovery (%)	RSD (%)
10	Podophyllotoxin	1–100	Y = 0.031207X+0.038612	0.9994	2.41	8.04	102.42±2.83	2.78
7	4’-Demethylpodophyllotoxin	1–100	Y = 0.024322X+0.044303	0.9991	1.93	7.86	103.61±3.12	3.16
8	4’-Demethylepipodophyllotoxin	1–100	Y = 0.039216X+0.065137	0.9995	2.15	8.01	101.23±2.69	2.73
15	Quercetin	0.5–50	Y = 0.012137X+0.006341	0.9996	2.92	9.88	98.25 ± 5.27	5.33
14	Kaempferol	0.5–50	Y = 0.021675X+0.018776	0.9993	3.07	10.23	98.68 ± 5.34	5.38

**Note:** LOD—Litnit of Detection, LOQ—Limit of quantitation, RSD—Relative standard deviation.

### Establishment and evaluation of the fingerprint of *Sinopodophyllum hexandrum* grown in different locations

The HPLC fingerprints of the eight production locations were established (Fig 2). Fig 2 shows that the relative retention time of every common peak in each of the HPLC fingerprints was consistent with that of the others, although the relative peak area differed significantly. The similarity of the LC fingerprint of each production location to the standard LC fingerprint S_0_ was evaluated by selecting different numbers of common peaks (5, 10, 15, 23) (Table 4). The evaluation results showed the lowest similarity (0.859–0.900) for *Sinopodophyllum hexandrum* from the Shangri-la, Yunnan, whereas the chemical fingerprints from other production locations had high similarity (above 0.900), indicating that the types of chemical ingredients contained in *S*. *hexandrum* roots and rhizomes were similar or the same among the eight production locations, but their contents exhibited significant differences.

**Table 4:** Similarity of chromatography of eight samples from eight locations.

Number of communal peaks	The similarities of eight samples								
	S_1_	S_2_	S_3_	S_4_	S_5_	S_6_	S_7_	S_8_	S_0_
5	0.944	0.912	0.924	0.946	0.933	0.859	0.919	0.963	1.000
10	0.971	0.966	0.963	0.955	0.947	0.868	0.982	0.956	1.000
15	0.975	0.968	0.964	0.954	0.947	0.869	0.983	0.957	1.000
23	0.982	0.970	0.966	0.952	0.953	0.900	0.986	0.961	1.000

**Note:** The cosine values of vectorial angle of the entire chromatographic patterns among different samples were calculated and the simulative standard chromatogram (S_0_) was set up with the median of all chromatograms using the Computer Aided Similarity Evaluation System for Chromatographic Fingerprint of Traditional Chinese Herbal Medicine (CASES), a chemometrics computer software endorsed by the National Pharmacopoeia Committee of the People’s Republic of China for similarity studies of chromatographic fingerprints of Chinese herbs. The cosine values of the two chromatograms (similarity) approaching 1 means they are highly similar.

### Differences in active ingredients in the roots and rhizomes of *Sinopodophyllum hexandrum* growing in different locations

**Fig 3:**
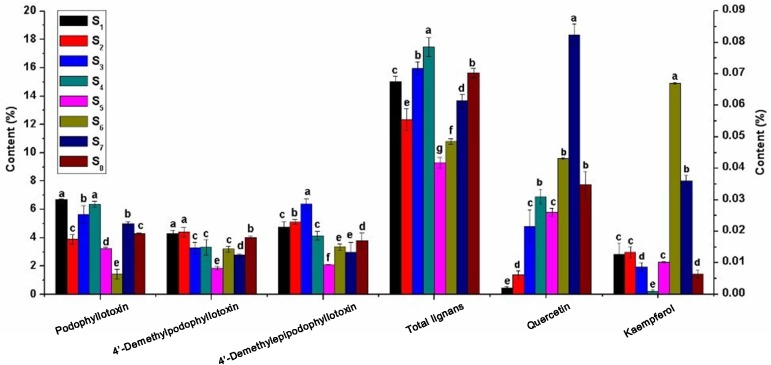
Differences in the contents of podophyllotoxin, 4’-Demethylpodophyllotoxin, 4’-Demethylepipodophyllotoxin, total lignans (see the left ordinate), quercetin and kaempferol (see the right ordinate) in the roots and rhizomes of *S*. *hexandrum* growing in different production locations. S_1_: Jingyuan, Ningxia; S_2_: Mei county, Shaanxi; S_3_: Huzhu, Qinghai; S_4_: Yongdeng, Gansu; S_5_: Kangding, Sichuan; S_6_: Shangri-la, Yunnan; S_7_: Nyingchi, Tibet; S_8_: Diebu, Gansu.

Significant differences were observed in the contents of the active ingredients of the roots and rhizomes of *Sinopodophyllum hexandrum* from different production locations (Fig 3). The highest PTOX contents were observed in the roots and rhizomes of *S*. *hexandrum* collected from Jingyuan, Ningxia (6.71%), which was five times higher than that in Shangri-la, Yunnan (the lowest level, 1.44%). The total lignan contents were the highest (17.46%) in Yongdeng, Gansu, followed by Huzhu, Qinhai (15.94%), whereas the lowest level was observed in samples from Kangding, Sichuan (9.31%), which was not consistent with the PTOX content ordering. This finding is reasonable because total lignans is a general term for a large group of substances that consists of many chemical ingredients, and the total lignan contents are determined by the contents of PTOX as well as other related ingredients. The 4'-demethylpodophylltoxin and 4'-demethyl-epipodophylltoxin contents were the highest in Mei county, Shaanxi (4.40%), and Huzhu, Qinghai (6.38%), respectively, and the lowest in Kangding, Sichuan (1.85%, 2.10%). The quercetin and kaempferol contents were low in all locations, but their content differences among the different production locations were obvious. The quercetin contents were the highest (0.0824%) in Nyingshi, Tibet, being 40 times higher than the lowest level, which was found in Jingyuan, Ningxia (0.0022%). The highest kaempferol contents (0.067%) were observed in Mei county, Shaanxi, being 61 times higher than the lowest level, which was found in Yongdeng, Gansu (0.0011%).

Although the content of each active ingredient from the roots and rhizomes of *Sinopodophyllum hexandrum* had significant differences, the orders of the contents of these substances were quite similar in each production location. Total lignan ranked the first, PTOX, 4'-demethylpodophylltoxin and 4'-demethylepipodophylltoxin ranked the second, and quercetin and kaempferol ranked the last place. The consistency of this ordering across eight test locations indicated the germplasm resources stability of *S*. *hexandrum* populations selected, potentially relating to the genetic characteristics of *S*. *hexandrum*.

### Analysis of ecological factors influencing the active ingredients of *Sinopodophyllum hexandrum*

#### Correlation analysis

The synthesis and accumulation (secondary metabolism) of the active ingredients of medicinal plants is an extremely complex process affected by a series of ecological factors, comprising a multivariable system. Soil factors were significantly different owing to different production locations and climate factors changed with geographical conditions of different study sites, which caused the differences in terms of growth environment of *Sinopodophyllum hexandrum* (Table 2). CA showed that the active ingredient contents were correlated to all of the ecological factors studied but at different levels (Table 5). PTOX was significantly and positively correlated to the X_JMT_ (R = 0.972), indicating that the temperature during the growth period of *S*. *hexandrum* had a strong effect on PTOX synthesis, followed by X_pH_ (R = –0.963). X_OM_ exhibited a highly significant and negative correlation with 4’-demethylpodophyllotoxin (R = –0.968), whereas the X_JMT_ demonstrated significant and positive correlation (R = 0.953). The X_FFP_ was significantly and positively correlated with 4’-demethylepipodophyllotoxin (R = 0.988) but significantly and negatively correlated with kaempferol (R = –0.967). Quercetin was significantly and positively correlated to pH (R = 0.969) but significantly and negatively correlated to X_AAT_ (R = –0.953). Total lignan contents were significantly and positively correlated to X_JMT_ (R = 0.978) but significantly and negatively correlated to X_RH_ (R = –0.964).

**Table 5:** The correlation analysis between ecological factors and the active ingredients.

Items	X_RAN_	X_RAP_	X_RAPM_	X_TN_	X_TP_	X_TPM_	X_OM_	X_pH_	X_JAT_	X_JMT_	X_AAT_	X_AHT_	X_ALT_	X_AMT_	X_AAP_	X_ASD_	X_FFP_	X_RH_	Y_P_	Y_DP_	Y_DEP_	Y_TL_	Y_Q_	Y_K_
X_RAN_	1																							
X_RAP_	-0.455	1																						
X_RAPM_	0.437	-0.442	1																					
X_TN_	-0.516	0.816	0.441	1																				
X_TP_	-0.332	0.848	0.372	0.993**	1																			
X_TPM_	0.513	0.834	0.417	0.872	0.750	1																		
X_OM_	-0.496	-0.667	-0.619	-0.514	-0.419	-0.633	1																	
X_pH_	-0.679	-0.487	0.499	0.447	-0.538	-0.546	0.408	1																
X_JAT_	0.834	-0.714	-0.485	-0.587	-0.498	0.195	-0.455	-0.794	1															
X_JMT_	0.788	0.411	-0.498	-0.485	-0.585	0.406	-0.544	-0.821	0.983**	1														
X_AAT_	0.618	-0.430	0.584	-0.410	-0.417	0.571	-0.851	-0.530	0.623	0.647	1													
X_AHT_	0.683	0.427	-0.439	-0.430	-0.519	0.637	-0.686	-0.949*	0.888*	0.945*	0.474	1												
X_ALT_	0.463	0.594	0.345	-0.427	-0.517	0.498	-0.833	-0.522	0.681	0.786	0.823	0.765	1											
X_AMT_	0.553	-0.607	-0.575	-0.438	-0.460	-0.425	0.595	-0.469	0.446	0.532	-0.547	0.501	-0.447	1										
X_AAP_	0.719	-0.542	-0.401	-0.518	-0.466	0.524	0.708	-0.84`	0.884*	0.831	0.493	0.795	0.527	0.797	1									
X_ASD_	-0.779	0.669	0.470	0.624	0.574	-0.518	0.813	0.753	-0.900*	-0.823	-0.415	-0.726	-0.499	-0.785	-0.983**	1								
X_FFP_	0.773	-0.524	-0.443	-0.630	-0.556	0.403	-0.431	-0.821	0.982**	0.951*	0.514	0.864	0.570	0.591	0.954*	-0.985*	1							
X_RH_	0.543	-0.514	-0.613	0.580	0.756	-0.803	0.831	-0.835	0.777	-0.878	0.546	0.734	-0.483	0.537	-0.883	0.782	0.724	1						
Y_P_	-0.438	-0.751	0.506	0.810	0.866	0.816	0.458	-0.963**	0.455	0.972**	-0.539	0.476	0.513	0.443	-0.902	0.934*	0.520	-0.438	1					
Y_DP_	0.632	0.833	0.693	0.347	0.542	0.776	-0.968**	0.423	0.695	0.953*	-0.733	0.814	0.724	0.446	0.716	0.915*	0.776	0.632	0.444	1				
Y_DEP_	0.782	0.490	0.591	0.579	0.556	0.473	0.514	0.889	0.732	0.575	-0.773	0.467	0.515	0.828	-0.847	0.930*	0.988**	0.480	0.554	0.667	1			
Y_TL_	0.799	-0.614	0.535	0.779	0.706	0.363	-0.950*	0.969**	-0.703	-0.896*	-0.953*	0.707	0.631	0.456	0.798	0.918*	0.641	0.538	0.643	0.823	0.553	1		
Y_Q_	0.654	0.878	0.765	0.882	0.872	0.711	0.553	-0.907*	-0.575	-0.438	-0.460	-0.425	0.595	-0.469	0.446	0.932*	-0.967**	0.955**	-0.438	-0.460	-0.425	0.654	1	
Y_K_	0.731	0.786	0831	0,869	0.843	0.738	-0.933	*0.635	-0.478	0.978**	-0.524	-0.603	0.610	-0.565	0.536	0.881	0.907*	-0.964**	0.501	0.570	-0.534	0.547	0.436	1

**Note:** * Significant at *P* < 0.05

** Significant at *P* < 0.01.

X_RAN_, X_RAP_, X_RAPM_, …, X_RH_ was performed in the Table 2, respectively. Y_P_-Podophyllotoxin, Y_DP_-4’-Demethylpodophyllotoxin, Y_DEP_-4’-Demethylepipodophyllotoxin, Y_TL_-Total lignans, Y_Q_-Quercetin, Y_K_-Kaempferol.

In summary, active ingredients are determined by the specific ecological conditions of their growing locations. Some complexity existed between the ecological factors and active ingredients. It was difficult to identify the dominant ecological factors that influence the six active ingredients of *Sinopodophyllum hexandrum* through CA.

#### Principal components analysis

PCA was performed to search for a principal component that is representative of the six types of active ingredients (Table 6). The contribution rate reflected the amount of original information contained within each factor. The first two principal components had higher eigenvalues, and their accumulated contribution rates reached 95.316% (Table 6), indicating that the first two main components included nearly all of the information of the six original components. Therefore, they could be extracted to obtain the loading matrix by orthogonal rotation (Table 7). The equation for the first principal component F_1_ was F_1_ = 0.959Y_P_ + 0.432Y_DP—_0.379Y_DEP_ + 0.974Y_TL_ + 0.878Y_Q_ + 0.912Y_K_, whereas that of the second principal component F_2_ was F_2_ = 0.246Y_P_ + 0.921Y_DP_ + 0.876Y_DEP_ + 0.057Y_TL_ + 0.321Y_Q—_0.221Y_K._ F_1_ accounted for a large proportion of Y_P_ (0.959), Y_TL_ (0.974), Y_Q_ (0.878), and Y_K_ (0.912) and accounted for 86.776% of all principal components. In contrast, F_2_ accounted for a large proportion of Y_DP_ (0.921) and Y_DEP_ (0.876), but its contribution rate only accounted for 8.54% of all principal components. Therefore, F_1_ was selected as the principal component to represent the active ingredients present in the roots and rhizomes of *Sinopodophyllum hexandrum*.

**Table 6:** Eigenvalues and cumulative contribution rates of principal components.

Principalcomponents	Eigenvalues	Contributionrates (%)	Cumulativecontribution rates (%)
F_1_	4.860	86.776	86.776
F_2_	1.553	8.54	95.316
F_3_	0.338	2.337	97.653
F_4_	0.127	1.418	99.071
F_5_	0.052	0.653	99.724
F_6_	0.022	0.276	100

**Note:** All the original data were standardized.

**Table 7:** Load matrix of the principal components.

Active ingredients	Load	
	The first principal component (F_1_)	The second principal component (F_2_)
Y_P_	0.959	0.246
Y_DP_	0.432	0.921
Y_DEP_	-0.379	0.876
Y_TL_	0.974	0.057
Y_Q_	0.878	0.321
Y_K_	0.912	-0.221

**Note:** Y_P_-Podophyllotoxin, Y_DP_-4’-Demethylpodophyllotoxin, Y_DEP_-4’-Demethylepipodophyllotoxin

Y_TL_-Total lignans, Y_Q_-Quercetin, Y_K_-Kaempferol.

#### Gray correlation analysis

GCA was conducted to deduce primary and secondary factors among the ecological factors based on gray correlation degree between the active ingredient contents and the ecological factors in this study [55,56]. The results of the analysis (Table 8) demonstrated that different ecological factors exerted different degrees of influence on the active ingredients: the primary ecological factors that affected the active ingredient contents were X_AAP_, X_JMT_, X_FFP_, X_ASD_, X_pH_, X_OM_, and X_RAPM_ (gray correlation degree ≥ 0.700), whereas the secondary ecological factors were X_AAT_, X_RH_, and X_AMT_ (gray correlation degree < 0.700).

**Table 8:** Gray correlation coefficients between the principal components and ecological factors.

Ecological factors	Gray correlation coefficients	Ecological factors	Gray correlation coefficients
X_RAN_	0.474	X_JMT_	0.832
X_RAP_	0.402	X_AAT_	0.667
X_RAPM_	0.723	X_AHT_	0.520
X_TN_	0.423	X_ALT_	0.554
X_TP_	0.316	X_AMT_	0.618
X_TPM_	0.357	X_AAP_	0.854
X_OM_	0.756	X_ASD_	0.789
X_pH_	0.778	X_FFP_	0.807
X_JAT_	0.579	X_RH_	0.643

**Note:** X_RAN_, X_RAP_, X_RAPM_, …, X_RH_ was performed in the Table 2, respectively.

On the other hand, although the primary ecological factors can be clarified by GCA, the correlations between the active ingredients and different primary ecological factors were still unclear.

#### Path analysis

A path analysis was carried out between the principal component (F_1_) and the primary ecological factors, including X_AAP_, X_JMT_, X_FFP_, X_ASD_, X_pH_, X_OM_, and X_RAPM_, which was used to express the correlation degree between them [57]. Most of the primary factors had negative and direct effects on the contents of the active ingredients (Table 9). The indirect effect of the primary factors on active ingredients was generally weaker than their direct effect, except for X_RAPM_ and X_OM_, indicating that the ecological factors screened by GCA played a direct and decisive role in the synthesis and accumulation of the active ingredients. X_pH_ had the most negative direct effect (- 0.9936) of all of the primary ecological factors but the lowest correlation coefficient (0.1157; the lowest significance level) with the active ingredients because its negative direct effect was counterbalanced by the positive indirect effects of X_FFP_, X_AAP_, X_JMT_, X_OM_, X_ASD_, and X_OM_. In terms of indirect effects, X_OM_ had a larger contribution (0.9961) than any other factor and mainly had a positive and indirect effect through X_RAPM_, X_AAP_, and X_JMT_ in the accumulation of the active ingredients, weakening their negative and direct effects. Thus, significant and positive correlations (*P* < 0.05) were observed between X_OM_ and the active ingredients. Obviously, a stronger direct effect between the primary ecological factors and the active ingredients did not always imply a higher correlation between them. The statistical significance (Table 9) in this study demonstrated that the higher the X_AAP_ and X_JMT_, the lower the contents of the active ingredients, and that the longer the X_ASD_ and the X_FFP_ and the higher the X_OM_, the higher the contents of the active ingredients. At the same time, the results of ANOVA analysis (Table 10) was significant (*F* = 35.268, *P*<0.01), which confirmed the availability and rationality of PA. The action pathway between the ecological factors and the principal components (F_1_) is complicated. Thus, we can’t be sure which factors play a decisive role in the production of the active ingredients among the seven primary ecological factors using PA. Further analyses were conducted to identify the determinant factors and their comprehensive decisive degree.

**Table 9:** Path analysis between the contents of active ingredients and primary ecological factors.

F	*P*	C	D					ID			
				Total	→X_RAPM_	→X_OM_	→X_pH_	→X_JMT_	→X_AAP_	→X_ASD_	→X_FFP_
X_RAPM_	0.2341	0.1769	-0.7983	0.9752		-0.0115	0.4381	0.3913	0.6301	-0.1405	-0.3323
X_OM_	0.0353	0.8087	-0.1874	0.9961	0.7686		-0.3205	0.3212	0.4535	-0.1011	-0.1256
X_pH_	0.3040	-0.1157	-0.9936	0.8779	-0.2306	0.2676		0.3532	0.3606	-0.3085	0.4356
X_JMT_	0.0062	-0.9179	-0.4609	-0.457	-0.4657	-0.3229	0.324		0.2525	0.3028	-0.5477
X_AAP_	0.0002	-0.9616	-0.6753	-0.2863	-0.8286	-0.0076	0.1021	0.3246		0.1087	0.0145
X_ASD_	0.0124	0.9007	0.9331	-0.0324	0.0867	-0.2281	0.0811	0.1507	0.0228		-0.1456
X_FFP_	0.0273	0.8468	0.7626	0.0842	0.3117	0.0262	0.071	-0.1255	0.0456	-0.2448	

**Note:** F-Factors; *P*-*P* value; C-Correlation coefficients; D-Direct path coefficients; ID-Indirect path coefficients. Absolute value of path coefficients (direct and indirect path coefficients) were used for analysis.

**Table 10:** Analysis of variance (ANOVA) for the contents of active ingredients and seven primary ecological factors.

Model	Sum of squares	df	Mean square	F value	Significance
Regression	711.991	7	101.713	35.268	0.003
Residual	138.432	48	2.884		
Total	850.423	55	104.597		

#### Analysis of decisive degree

The determinant factors and comprehensive decisive degree were assessed using the determination coefficient in analysis of decisive degree (ADD) [58]. The determination coefficient R_(i)_ (determination ability) of each factor X_i_ (independent variable) to Y_i_ (dependent variable) was expressed by the formula and calculated on basis of PA data as well as the multivariate determination coefficient R_0_^2^ (the total determination ability) and the determination coefficient *P*_e_ of those unconsidered factors in Table 11 in the present polyfactorial system that contained many factors and interaction effects.

**Table 11:** Determination coefficients of primary ecological factors.

Factors	Related formulas and computations
	${\text{R}}_{\left(\text{i}\right)}={\text{P}}_{\text{i}}{}^{\text{2}}+\text{2}\sum_{\text{k}\neq \text{i}}{\text{P}}_{\text{i}}{\text{P}}_{\text{ik}}={\text{d}}_{\text{i}}+\sum_{\text{k}\neq \text{i}}{\text{d}}_{\text{ik}}=2{\text{P}}_{\text{i}}{\text{r}}_{\text{iy}}\frac{}{}-{\text{d}}_{\text{i}}$
	${\text{R}}_{0}^{2}=\sum_{\text{i}=\text{1}}^{\text{m}}{\text{d}}_{\text{i}}+2\sum_{\text{i}<\text{j}}{\text{P}}_{\text{i}}{\text{P}}_{\text{ij}}$
	${\text{P}}_{\text{e}}=\sqrt{1{\text{-R}}_{0}^{2}}$
X_RAPM_	R_(RAPM)_ = 2 × (- 0.7983) × (0.1769) – (- 0.7983)^2^ = – 0.9197
X_OM_	R_(OM)_ = 2 × (- 0.1874) × (0.8087) – (- 0.1874)^2^ = - 0.3382
X_pH_	R_(pH)_ = 2 × (- 0.9936) × (- 0.1157) – (- 0.9936)^2^ = - 0.7573
X_JMT_	R_(JMT)_ = 2 × (- 0.4609) × (- 0.9179) – (- 0.4609)^2^ = 0.6337
X_AAP_	R_(AAP)_ = 2 × (- 0.6753) × (- 0.9616) – (- 0.6753)^2^ = 0.8427
X_ASD_	R_(ASD)_ = 2 × (0.9331) × (0.9007) – (0.9331)^2^ = 0.8102
X_FFP_	R_(FFP)_ = 2 × (0.7626) × (0.8468) – (0.7626)^2^ = 0.7100
R_0_^2^	R_0_^2^ = 3.7803 + 2 × (- 1.3995) = 0.9813
P_e_	${\text{P}}_{\text{e}}=\sqrt{1\text{−}0.9813}$ = 0.1367
R_(AAP)_ >R_(ASD)_ >R_(FFP)_ >R_(JMT)_ >0 >R_(OM)_ >R_(pH)_ >R_(RAPM)_	

**Note:** i, j = 1, 2, …, m; P_i_, direct path coefficient; P_ij_, total of indirect path coefficients; r_iy_, correlation coefficient between X_i_ and Y;

d_i_ = P_i_^2^; see Table 9. R_(i)_, determination coefficient (determination ability) of each factor X_i_ (independent variable) to Y_i_ (dependent variable); R_0_^2^, the multivariate determination coefficient (the total determination ability) of the seven primary ecological factors; P_e_, determination coefficient of those unconsidered factors.

The determination coefficients for X_JMT_, X_FFP_, X_ASD_, and X_AAP_ were positive, indicating that these four ecological factors had strong and comprehensive influences on the active ingredient contents of *Sinopodophyllum hexandrum* and can be considered important determinant factors. The X_AAP_ was the most important determinant factor, as R_(AAP)_ value was the highest. The determination coefficients of X_RAPM_, X_pH_, and X_OM_ were negative, indicating that they were important limiting factors. Compared with the soil factors (X_RAPM_, X_pH_, and X_OM_), climate factors (X_JMT_, X_FFP_, X_ASD_, and X_AAP_) had a stronger influence on the contents of the active ingredients in the roots and rhizomes of *S*. *hexandrum*. This finding indicated the importance of controlling climate conditions in the production process of *S*. *hexandrum* to produce high-quality medicinal materials for the treatment of human diseases.

## Discussion

### Variation in active ingredient contents

The metabolism and accumulation of active ingredients are strongly affected, either directly or indirectly, by environmental factors. Most plants regulate the types and amount of active ingredients according to environmental variations. In this study, significant differences were observed in the active ingredient contents in the roots and rhizomes of *Sinopodophyllum hexandrum* planted at different production locations (Fig 3), whereas insignificant differences were noted in the types of constitutes (Fig 2, Table 4). These results were consistent with previous reports [15]. The results indicated that the active ingredient contents were closely related to the growing locations of *S*. *hexandrum* and environmental factors thereof influenced the production of active ingredients.

The contents of PTOX in the roots and rhizomes of *Sinopodophyllum hexandrum* were correlated with latitude and altitude to some degree. The higher the latitude (northerly) and the lower the altitude, the higher the contents of PTOX, and the lower the latitude (southerly) and the higher the altitude, the lower the contents of PTOX (Figs 1 and 3, Table 1). For example, the PTOX contents in the roots and rhizomes of *S*. *hexandrum* planted in Jingyuan, Ningxia (northerly, 2 488 m), and Yongdeng, Gansu (northerly, 2 500 m), were higher than those planted in Shangri-La, Yunnan (southerly, 3300 m) and Nyingchi, Tibet (southerly, 3100 m). The trend for quercetin and kaempferol contents was opposite to that of PTOX, being the highest in Nyingchi, Tibet, and Shangri-La, Yunnan, respectively. Interestingly, the altitude of these two production locations was also high. Quercetin and kaempferol present an ortho-dihydroxylated structure, exhibiting ultraviolet absorption and free-radical scavenging, allowing the *S*. *hexandrum* plant to grow healthily under the strong ultraviolet radiation in higher altitude areas [59–61]. Altitude and latitude, two important factors that affect the hydrothermal conditions of plant growth environment, have important effects on plant secondary metabolic processes. Thus, the causes of the correlation between the active ingredient contents and these two factors in their growing locations require further study by adopting molecular biology methods associated with ecological factors, and reproductive and vegetative factors.

### Precipitation and active ingredient contents

In this study, ADD revealed that the X_AAP_ was the most important determinant factor (Table 11). At the same time, GCA and PA also showed that the strongest negative correlation existed between X_AAP_ and the contents of the active ingredients (Tables 8 and 9). This finding was consistent with the previous research results about other active substances. For example, the quinine content of *Cinchona ledgeriana* (Howard) Bern. Moens ex Trimen was higher under high-temperature and drought conditions and significantly lower under excessive soil humidity, even to the point of not being synthesized at all. The atropine content of *Anisodus carniolicoides* (C.Y. Wu & C. Chen) D'Arcy & Zhi Y. Zhang could be as high as 1% under dry conditions, which is 2.5 times greater than that obtained under humid conditions (0.4%). The high soil moisture content sharply decreased the alkaloid contents of *Ephedra intermedia* Schrenk ex C.A. Mey., which increased under dry conditions [62]. By contrast, the active ingredient contents of some medicinal plants, such as the ihapine glucosinalbate content of *Sinapis alba* L., increased with increased precipitation [63].

### Sunshine duration and active ingredient contents

The illumination can affect the active ingredient contents of medicinal plants [62]. To some plants, the increase of illumination time can increase the contents of active ingredients. Dong et al. reported the influence of environmental factors on the contents of secondary metabolites in the leaves of *Eucommia ulmoides* Oliv. and found that annual sunshine duration was significantly and positively correlated to the contents of geniposidic acid (*P*< 0.05) [37]. Zhu *et al*. [64] found that the total saponin contents of *Panax quinquefolius* L. increased linearly by 6.75% to a maximum of 8.72% with annual sunshine duration in the altitude range of 530 m to 850 m. Meanwhile, the total saponin contents dramatically decreased because of the sharp reduction in annual sunshine duration caused by the existence of a strong fog and cloud belt in the altitude range of 850 m to 1000 m, decreasing by 1.38% for every 100 m. Fuglevand et al. also confirmed that the amounts of flavonoids in *Arabidopsis* (DC.) Heynh. increased after long time illumination [59]. *Sinopodophyllum hexandrum* is a heliophilous plant, thus, locations with long sunshine durations would be favorable for its secondary metabolism, resulting in an adequate substrate to catalyze the synthesis of the active ingredients and thereby increasing their contents. PA and ADD showed that the active ingredient contents were highly associated with sunshine duration, with a significant and positive correlation (Tables 9 and 11), indicating that annual sunshine duration (X_ASD_) was an important ecological factor for the synthesis and accumulation of the active ingredients in the roots and rhizomes of *S*. *hexandrum*.

### Temperature and active ingredient contents

The production of secondary metabolites due to temperature stress is an expression of the self-defense mechanism of medicinal plants. Environmental temperature significantly affects the contents of secondary metabolites in plants [65]. For example, the hypericin contents in *Hypericum perforatum* L. increased with temperature [65], as did the contents of anthocyanin in sugarcane [66]. In the leaves of *Eucommia ulmoides* Oliv., annual average temperature was significantly and positively correlated to the contents of chlorogenic acid and flavonoids (*P*< 0.05) [37]. Similarly, PA showed that the contents of active ingredients were significantly and positively correlated with july mean temperature (X_JMT_) in this study (Table 9), but with the lowest positive decisive degree of all factors (Table 11). This was possibly related to the chemical structure of the active ingredients, which can easily decompose at high temperature.

### Soil and active ingredient contents

The active ingredient contents and the quality of medicinal plants are closely related to soil fertility [62]. As observed from the sequence of determination coefficients of the seven primary ecological factors, X_RAPM_ was important limiting factor, followed by X_pH_. However, these factors were not significantly correlated with the active ingredient contents at 0.05 (Table 9). Thus, X_OM_, with a positive and significant correlation to the active ingredient contents, was considered as the most important limiting factor. This result agreed with the findings for other active ingredients involved in medicinal plants. For example, Liu *et al*. [67] found that the saponin contents of *Panax quinquefolius* L. were increased by 27.86% by the repeated applications of high-quality organic fertilizer and that the functionality of potassic fertilizer was the same as that of a total nutrient admixture in terms of saponin contents. These findings were similar to those for the active ingredients in *Sinopodophyllum hexandrum*. Notably, although X_pH_ had the strongest direct effect on the active ingredient contents (Table 9), its decisive degree was not high (Table 11) because the accumulation and synthesis of the active ingredients was an overall reflection of the interaction of multiple ecological factors, such as temperature, humidity, and solar radiation.

### *Sinopodophyllum hexandrum* geo-authenticity

The quality of medicinal herbs is closely related to the local growth environment. Differences in production location would lead to differences in ecological factors, such as precipitation, illumination, temperature, humidity, and soil (Table 2), which would in turn lead to variation in the contents of active ingredients in this medicinal plant (Fig 3). The final result is differences in the medicinal quality and therapeutic effects of these plants. The quality of medicinal plants is specific to their environmental conditions, which corresponds to the concept of geo-authentic herbal drugs developed based on the experience of ancient people. However, these conditions do not always hold because the ancient people judged the quality of medicinal drugs only by their external quality and their medical experience. They were unaware of so called active chemical ingredients in medicinal plants [37]. The geo-authentic region of *S*. *hexandrum* has yet to be elucidated. In the present research, the key finding was that R_0_^2^ of the seven primary ecological factors was 0.9813, whereas the determination coefficient *P*_e_ of those unconsidered factors was only 0.1367 (Table 11). This finding demonstrated that the effect of ecological factors on the geographical variation of the active ingredient contents in roots and rhizomes of *S*. *hexandrum* was up to 98.13%, implying that the ecological factors explained 98.13% of the geographical variation of the active ingredient contents, which was consistent with previous studies [42,43]. Therefore, the results provided reliable reference data upon which to base decisions regarding understanding the influence of ecological factors on the variation of phytochemicals in *S*. *hexandrum* and selection of geo-authentic regions for yielding optimal medicinal raw materials. *S*. *hexandrum* is native to the Himalayan region, including China, India, Nepal, Myanmar, Afghanistan and Pakistan. No other chemical ecology studies of *S*. *hexandrum* exist in other locations. A comprehensive and systematic study including more populations in other regions of China and countries combined with molecular ecology and physiological ecology is required in a future study.

## Conclusions

The ecological factors significantly affected the active ingredient contents of the roots and rhizomes of *Sinopodophyllum hexandrum*, which contributed to their significant geographical differences in different production locations throughout China. The primary ecological factors affecting the active ingredient contents included annual average precipitation, July average temperatures, frost-free period, annual sunshine duration, soil pH, organic matter, and rapidly available potassium. The annual average precipitation was the most important determinant factor, being significantly and negatively correlated with the active ingredients (*P* < 0.001). A significant and positive correlation was observed between the annual sunshine duration and frost-free period and the active ingredient contents, whereas a significant and negative correlation was observed between July average temperature and the active ingredient contents. Organic matter, soil pH, and rapidly available potassium were the limiting factors; specifically, organic matter was the most important limiting factor, with a positive and significant correlation with the active ingredient contents. The selection of favorable production locations should be prioritized and the collection was scientifically carried out under the permission of local management departments for sustainable collection when attempting to harvest high-quality wild resources of *S*. *hexandrum*. Integrating the findings for all of the active ingredients and the ecological factors of each production location, it was concluded that Jingyuan in Ningxia Province and Yongdeng in Gansu Province were favorable production locations for *S*. *hexandrum* containing satisfactory PTOX and lignans and that Shangri-La in Yunnan Province and Nyingchi in Tibet were favorable for the production of *S*. *hexandrum* containing satisfactory quercetin and kaempferol contents.

## Supporting Information

S1 Table The names of the authorities who issued the permission for each location. (DOCX)

S2 Table The names of local meteorological bureaus (stations) for each location. (DOCX)

S1 Text Description of the sampling procedures. (DOC)
