# Supplementary material for: Influence of Ecological Factors on the Production of Active Substances in the Anti-Cancer Plant Sinopodophyllum hexandrum (Royle) T.S. Ying
Source: PLoS One. 2015 Apr 15;10(4):e0122981. doi: 10.1371/journal.pone.0122981 (PMC4398539; doi:10.1371/journal.pone.0122981)
Supplement: S2 Table — (DOCX) [file pone.0122981.s002.docx]

**Influence of Ecological Factors on the Production of Active Substances in the Anti-cancer Plant *Sinopodophyllum hexandrum* (Royle) T.S. Ying**

(Supporting Information S2 Table)

Wei Liu, Jianjun Liu^*^, Dongxue Yin, Xiaowen Zhao

College of Forestry, Northwest A & F University, Yangling, China

* E-mail: ljj@nwsuaf.edu.cn

**S2 Table. The names of local meteorological bureaus (stations) for each location.**

| No. | Study sites | Population | Code | Coordinates | Altitude (m) | Affiliation names | Address |
| --- | --- | --- | --- | --- | --- | --- | --- |
| S_1_ | Jingyuan, Ningxia | Baiyunshan | BYS | E106°15′N35°37′ | 2232 | Yehe valley meteorological stations | Mountain Liupan, Jingyuan county, 756400, Ningxia province. |
|  |  | Yehegu | YHG | E106°13′N35°31′ | 2370 |  |  |
|  |  | Zhiwuyuan | ZWY | E106°18′N35°22′ | 2080 |  |  |
|  |  | Qiaozigou | QZG | E106°22′N35°15′ | 2564 |  |  |
| S_2_ | Mei county, Shaanxi | Pingansi | PAS | E107°43′N34°1′ | 2815 | Haoping meteorological station | Yingtou town, Mei county, 722300, Shaanxi province. |
|  |  | Mingxingsi | MXS | E107°44′N34°0′ | 2637 |  |  |
|  |  | Yuhuangmiao | YHM | E107°22′N34°5′ | 1780 |  |  |
|  |  | Liulingou | LLG | E108°10′N33°52′ | 1013 |  |  |
| S_3_ | Huzhu, Qinghai | Zhalongkou | ZLK | E102°34′N36°53′ | 2264 | Meteorological station, Huzhu Northern Mt. | Jiading town, Huzhu county, 810500, Qinghai province. |
|  |  | Zhalonggou | ZLG | E102°37′N36°47′ | 2698 |  |  |
|  |  | Yuanlongogu | YLG | E102°27′N36°54′ | 3069 |  |  |
|  |  | Xiahe | XH | E102°42′N36°44′ | 3169 |  |  |
| S_4_ | Yongdeng, Gansu | Suoergou | SEG | E102°43′N36°40′ | 2389 | Xiaotugou meteorological station | Liancheng town, Yongdeng county, 730300, Gansu province. |
|  |  | Lalagou | LL | E102°43′N36°35′ | 2733 |  |  |
|  |  | Dachang | DC | E102°44′N36°44′ | 2449 |  |  |
|  |  | Datanzigou | DTZ | E102°46′N36°33′ | 2530 |  |  |
| S_5_ | Kangding, Sichuan | Yajaigeng | YJG | E101°57′N30°0′ | 2946 | Kangding county meteorological bureau | No. 31 east street, Kangding county, 626000, Sichuan province. |
|  |  | Laoyulin | LYL | E101°59′N29°55′ | 3788 |  |  |
|  |  | Shengkangcun | SKC | E102°1′N30°4′ | 3207 |  |  |
|  |  | Zhonggucun | ZGC | E101°54′N30°16′ | 3554 |  |  |
| S_6_ | Shangri-la, Yunnan | Rime | RM | E99°37′N27°51′ | 3528 | Shangri-la meteorological bureau | No.10 Changdu Road, Jiantang town, Shangri-la, 674400, Yunnan province. |
|  |  | Naipi | NP | E99°36′N28°2′ | 3432 |  |  |
|  |  | Xiaozhongdian | XZD | E99°56′N27°28′ | 3590 |  |  |
|  |  | Mugaocun | MGC | E99°34′N27°30′ | 2250 |  |  |
| S_7_ | Nyingchi, Tibet | Zhangmaicun | ZMC | E94°20′N29°40′ | 3097 | Nyingchi county meteorological bureau | Bayi town, Nyingchi county, 860100, Tibet. |
|  |  | Selong | SL | E94°11′N29°44′ | 3173 |  |  |
|  |  | Pula | PL | E94°22′N29°27′ | 3256 |  |  |
|  |  | Duosongba | DSB | E94°13′N29°37′ | 3855 |  |  |
| S_8_ | Diebu, Gansu | Zemo | ZM | E103°21′N33°45′ | 2728 | Ladagou meteorological station | Ladagou, Diebu county, 747400, Gansu province. |
|  |  | Dalong | DL | E103°14′N35°2′ | 2620 |  |  |
|  |  | Dalagou | DLG | E103°22′N33°52′ | 2677 |  |  |
|  |  | Nagai | NG | E103°14′N33°51′ | 2963 |  |  |
