# Supplementary material for: Influence of Ecological Factors on the Production of Active Substances in the Anti-Cancer Plant Sinopodophyllum hexandrum (Royle) T.S. Ying
Source: PLoS One. 2015 Apr 15;10(4):e0122981. doi: 10.1371/journal.pone.0122981 (PMC4398539; doi:10.1371/journal.pone.0122981)
Supplement: S1 Text — (DOC) [file pone.0122981.s003.doc]

**Influence of Ecological Factors on the Production of Active Substances in the Anti-cancer Plant *Sinopodophyllum hexandrum* (Royle) T.S. Ying**

(Supporting Information S1 Text)

Wei Liu, Jianjun Liu*, Dongxue Yin, Xiaowen Zhao

College of Forestry, Northwest A & F University, Yangling, China

* E-mail: ljj@nwsuaf.edu.cn

**Description of the sampling procedures**

*Sinopodophyllum hexandrum* (Royle) T.S. Ying has been classified as an endangered species (grade 3) since 1987 and catalogued in the Chinese Plant Red Book. Currently, with the enhanced awareness of its medicinal value and superior efficacy in clinical applications, the availability of *S. hexandrum* resource has become increasingly limited due to intense collection and the lack of organized cultivation in China. This work was supported by the program from the Forestry Research Foundation for the Public Service Industry of China (200904004). Its aims to establish management strategies for rational exploitation and conservation of *S. hexandrum* resources rather than randomly and extensively harvesting wild resources. Thus, specific permissions were not required for the described field sampling studies or for the collection of plant materials. Firstly, the authority of Northwest A & F University of issued a certificate of the investigation and research for each site. Secondly, the local management department checked the information and issued “Enter Proof”. Thirdly, we entered into the study sites for sampling.
